# A first-principles study on potential chelation agents and indicators of Alzheimer's disease

**DOI:** 10.1039/d0ra06855a

**Published:** 2020-09-28

**Authors:** Bryan Wang, Xuan Luo

**Affiliations:** National Graphene Research and Development Center Springfield Virginia 22151 USA

## Abstract

Human-serum transferrin is involved in the transportation of aluminum across the blood–brain barrier. Aluminum accumulation within the neuron causes the cell to degrade. In our research, we considered 12 potential chelators of aluminum from the aluminum–human serum transferrin complex and 3 potential indicators of Alzheimer's. We performed Density Functional Theory calculations comparing the binding energies of aluminum–chelator complexes and the binding energy of the aluminum–human serum transferrin complex and determined the charge transfer of the aluminum–chelator complex. Our results showed that CDTA is the only one that has direct chelation potential, but 1-ethyl-3-hydroxypyridin-2-one, citric acid, DTPA, oxalic acid, and salicylhydroxamic acid also had a strong and stable bond with aluminum and still have the ability to be potential chelators. The charge transfer calculation further enforces that these 6 chelators have strong and stable bonds with aluminum. Furthermore, we evaluated potential indicators of Alzheimer's disease. Metals that have a similar binding affinity to human serum transferrin as that of iron prove to be potential indicators of Alzheimer's disease. Due to the minimal difference in binding energies of the gallium–human serum transferrin complex and the indium–human serum transferrin complex to the iron–human serum transferrin complex, we determined that gallium and indium could be potential indicators of Alzheimer's disease.

## Abstract 2

Human-serum transferrin is involved in the transportation of aluminum across the blood–brain barrier.

## Graphical abstract

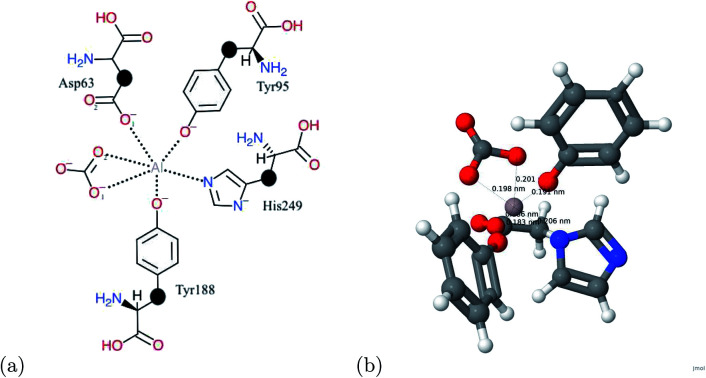

## I. Introduction

Neurological diseases affect millions of people throughout the world and have a variety of effects on humans including paralysis, muscle weakness, seizures, loss of sensation, blurry vision, poor cognitive abilities, unexplained pain, and decreased alertness.^1^ Common neurological diseases include Alzheimer's disease (AD), epilepsy, multiple sclerosis, Friedreich's ataxia, and Parkinson's disease.^1,2^ The study of neurological diseases was founded by Thomas Willis in the 1600s.^3^ On the molecular level, these neurological diseases are a result of the gradual breakdown and death of neurons.^2^ Current treatments include cell replacement therapy and gene transfer have provided the development of new therapeutic strategies for patients with neurological diseases such as Alzheimer's.^4^

The effects of AD cause a physical, financial, and emotional strain on both the AD-afflicted individual and the individuals family members.^5^ AD was first discovered in 1910, and the first drug developed to combat Alzheimer's was during the late 1980s.^6^ Now, it is one of the most common diseases, and one new person in America develops AD every 68 seconds.^7^ Since there is currently no cure for AD, current AD treatments only help mitigate the symptoms.^8^ Drugs such as donepezil and galantamine prevent the breakdown of acetylcholine, which improves mental functions including memory and attention.^8^

In the last few decades, there have been many studies on the impact of aluminum in the development of AD.^9,10^ Many experiments have been conducted revealing there is a correlation between increased aluminum content in the brain and AD.^9–12^ The content of aluminum in the brain increases with age for all individuals even for those who do not experience dementia.^13^ Aluminum exposure has increased dramatically in human lives in the past century in the forms of medicines, drinking water, and cookware, but aluminum does not have a biochemical role.^14^ From an evolutionary viewpoint, aluminum begins to accumulate within neurons because the body does not have a process to eliminate aluminum.^14,15^ Researchers have done experimental procedures and quantum mechanical calculations to analyze the effects of aluminum in the human body.^9,16^ It was found that almost 90% of the aluminum in the human body bind to human serum transferrin.^17^ Within the brain, aluminum is not evenly distributed, but rather it is primarily located in regions with a high transferrin receptor density.^18,19^

Human serum transferrin is an iron transport protein in vertebrates with a molecular weight of approximately 80 kDa.^20^ It belongs to a family of proteins that serve as bacteriostatic agents and cellular iron uptake.^21^ Transferrin consists of 2 lobes: N lobe and C lobe.^22,23^ Each of these lobes is further subdivided into domains of approximately equal size: N1, N2, C1, and C2.^22,24^ There is only one iron binding site which is in the cleft of the N and C site.^22^ When a metal binds to this binding site, the metal forms a distorted octahedral shape with the surrounding ligands.^21^ These binding ligands consist of one histidine, two tyrosines, one aspartic acid, and one bidentate carbonate ion.^24^ The carbonate ion proves to be an essential and defining trait of the transferrin protein, and no metal would be able to bind to the transferrin molecule if it were not present.^16^ Human serum transferrin transports aluminum across the blood–brain barrier, and as aluminum accumulates within neurons, it prevents proper conduction of stimuli and begins to degrade the neuron.^25^

To find a solution to this problem, we chose metals with similar properties to that of aluminum and iron such as gallium, indium, and chromium that can be potential indicators of AD. Gallium and indium are of great interest due to its use as radioactive isotopes in diagnosing patients.^21^ Furthermore, past studies have shown gallium to be a predictor of AD since it has a similar binding affinity with transferrin as iron.^26^ Additionally, we used chelators to remove aluminum from the binding site of human serum transferrin. The process of chelation utilizes compounds to reduce the concentration of metals in the human body. In doing so, it would lower the aluminum concentration within the brain and allow for proper signal conduction. This will hopefully help reduce the rate of AD. We carried out a computational calculation on replacing aluminum in serum transferrin with one of the metals from above, and we also tried to determine a potential solution to aluminum bound to transferrin through a chelator. Analysis of these effects probably will be useful in solving the cure for Alzheimer's and new methods of treatments.

## II. Method

In this study, we investigated the potential chelation of aluminum from the human serum transferrin binding site and potential indicators of AD by conducting a first principle methods study. The binding energy and charge transfer of the metal–molecule complexes are calculated to determine the most optimal chelator and indicator (Table 1).

**Table 1:** Electron configurations and radius cutoff of each element for generating the PAW pseudopotentials used in the current study

Element	Electron configuration	Radius cut-off (Bohr)
Hydrogen (H)	1s^1^	1.00
Carbon (C)	[He]2s^2^2p^2^	1.51
Nitrogen (N)	[He]2s^2^2p^3^	1.20
Oxygen (O)	[He]2s^2^2p^4^	1.41
Aluminum (Al)	[Ne]3s^2^3p^1^	1.90
Chromium (Cr)	[Ar]3d^5^4s^1^	2.11
Iron (Fe)	[Ar]3d^6^4s^2^	2.12
Gallium (Ga)	[Ar]3d^10^4s^2^4p^1^	2.10
Indium (In)	[Kr]4d^10^5s^2^5p^1^	2.51

### A. Computational details

Density Functional Theory (DFT)^27^ calculations employed Generalized Gradient Approximation (GGA) in the form of Perdew–Burke–Ernzerhof (PBE)^28^ implemented in the ABINIT code^29,30^ were used to perform first-principle calculations in this study. At the same time, GGA-PBE exchange–correlation functionals are also used to generate Projected Augmented Wave (PAW) pseudopotentials.^31^

In the self-consistent field (SCF) total energy calculations, the SCF iterations terminated once the total energy difference was less than 1.0 × 10^−8^ Ha twice consecutively. For the lattice parameter optimization calculations, the Broyden–Fletcher–Goldfarb–Shanno (BFGS) method was used for the relaxation of the atomic structure. The BFGS relaxation was considered converged once all the forces was less than 5.0 × 10^−3^ Ha Bohr^−1^. The SCF iterations were considered converged once the difference of total forces was less than 5.0 × 10^−5^ Ha Bohr^−1^ twice consecutively.

### B. Metal calculations

The primary calculation in this study was calculating the binding energy. To determine the binding energy, the total energies of the materials must be obtained first. However, in order to determine the total energy, a series of calculations including the convergence of the kinetic energy cutoff, the *K*-mesh, and optimized lattice parameter must be performed. The kinetic energy cutoff and *K*-mesh were converged when the total energy difference of the data sets were smaller than 0.0001 Ha twice consecutively. Using the converged values, the structural relaxation were performed using the BFGS method to determine the optimized lattice constants of the metals. After all structures were fully relaxed, the total energy was then calculated.

### C. Molecular and metal–molecule complex calculations

To determine the binding energy, the kinetic energy cut-off was determined for each atom of the system. The highest kinetic energy cut-off was then used. Using the kinetic energy cut-off, we performed a structural relaxation with the BFGS method to obtain the optimized coordinates of the molecule and the metal–molecule complex. After the molecule and the metal–molecule complex were fully relaxed, the total energy was then calculated with the converged values.

The initial coordinates of the human serum transferrin (1D3K in the Protein Data Bank) were obtained from the X-ray crystal structure.^32^ The metals used in this study were put in the original binding site of Fe in human serum transferrin. The hydrogen atom incorporation was performed at a pH of 7.5 for all titratable amino acids and was based on a previous paper.^16^ The entire system is composed of the metal and the first coordination sphere. The first coordination sphere is composed of a carbonate ion, 2 tyrosines (Tyr95, Tyr188), aspartic acid (Asp63), and histidine (His249). Using a unit cell with dimensions of 35 Bohr × 35 Bohr × 30 Bohr, the first coordination sphere and metal are first optimized in the Jmol software. The optimized structures coordinates are then exported and used in the relaxation calculations.

### D. Binding energy

The total energy of the molecule (first coordination sphere of transferrin/chelator) was calculated both with and without the metal. The binding energy can be obtained from the following equation

1 *E*_binding_ = *E*_metal+molecule_ − *E*_metal_ − *E*_molecule_

where *E*_binding_ represents the binding energy, *E*_metal+molecule_ is the total energy of the metal–molecule complex, *E*_metal_ is the total energy of the metal, and *E*_molecule_ is the total energy of the molecule. The higher the magnitude of the binding energy is associated with a higher affinity.

### E. Charge transfer

Potential chelator candidates were carried out to do charge transfer calculations. The charge transfer was based on three total energy calculations including metal ion, chelator, metal–chelator complex. Charge density differences were obtained with the following equation.

2 Δ*ρ* = *ρ*_metal+molecule_ − *ρ*_metal_ − *ρ*_molecule_

where Δ*ρ* represents the charge transfer between the metal and the molecule, *ρ*_metal+molecule_ represents the charge of the metal–molecule complex, *ρ*_metal_ represents the charge of the metal ion, and *ρ*_molecule_ is the charge of the molecule. The isosurface values displayed the charge densities where a positive isosurface value represents the gain of electrons and a negative isosurface value represents the loss of electrons.

## III. Results and discussion

In this section, we discuss the results of the converged values, the binding strength between different metals and human serum transferrin, and the chelation of aluminum from the transferrin binding site. Referring to eqn (1), the binding energies were calculated using the total energies of the metal ion, the molecule (first coordination sphere of transferrin/chelator), and the metal–molecule complex. After obtaining the binding energies, we analyzed and interpreted the meaning of the results in the context of the project: the chelation potential of the proposed treatments.

### A. Metal calculations

The kinetic energy cutoff, the *K*-mesh, and the optimized lattice parameter were calculated in this study and are presented in Table 2. The optimized lattice parameters and the experimental lattice parameters from previous studies are presented in Table 2. Based on the table, the optimized lattice parameters are similar to the experimental lattice parameters.

**Table 2:** Converged kinetic energy cut-off, *K*-mesh, and optimized and experimental lattice parameters in Å of the metals in the current study

Metal	Crystal structure	KE cut-off (Ha)	*K*-Mesh	Optimized (Å)	Experimental (Å)	Percent Error
Al	FCC	12	12 × 12 × 12	4.05	4.05	0.06%
Cr	BCC	22	8 × 8 × 8	2.85	2.91	2.27%
Fe	BCC	23	8 × 8 × 8	2.76	2.87	3.95%
Ga	Orthorhombic	18	16 × 16 × 16	4.52	4.52	0.05%
In	BCC	21	10 × 10 × 10	3.37	3.25	3.48%

### B. Metal–molecule complex calculations

#### 1. Human serum transferrin interaction with metal

**Fig. 1:**
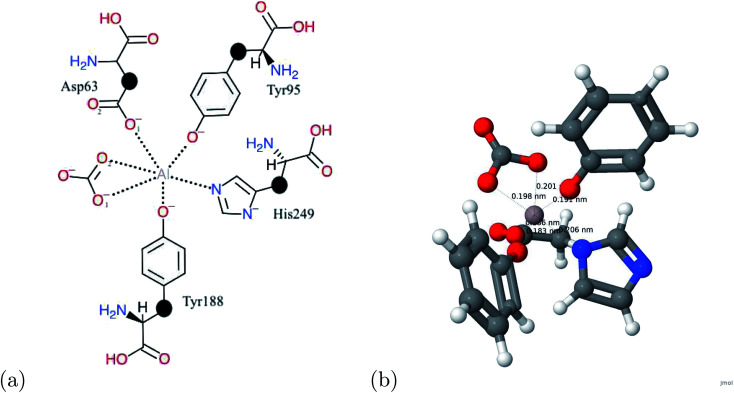
(a) A schematic view of the transferrin binding site. The link atoms are represented by •. (b) The atomic structure of transferrin and aluminum. For the atomic structure, the carbon, hydrogen, oxygen, nitrogen, and aluminum atoms are represented by gray, white, red, blue, and grayish red respectively.

**Fig. 2:**
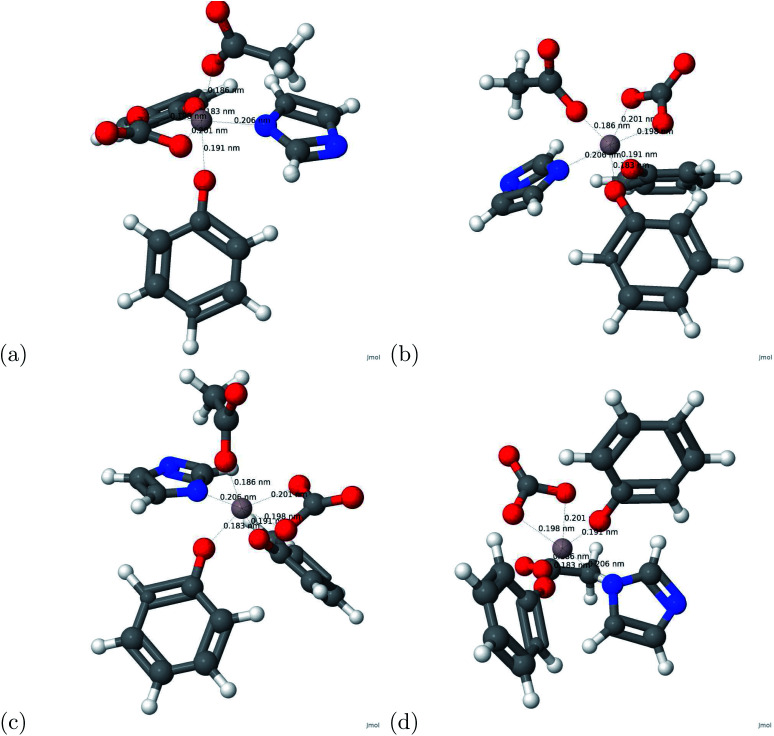
The optimized structure of the Al–transferrin complex. For the atomic structure, the carbon, hydrogen, oxygen, nitrogen, and aluminum atoms are represented by gray, white, red, blue, and grayish red respectively.

The calculation regarding metals and human serum transferrin was subject to computational limits. To accommodate for the computational limits, the system consisted of the metal and the first coordination sphere of human serum transferrin accompanied with the link atom method refer to Fig. 1. Optimized atomic coordinates were determined from the relaxation calculations (Table 3 and Fig. 2).

**Table 3:** The bond lengths (Å) between each metal and the ligands of the first coordination sphere of human serum transferrin in the current study

Metal	NHis249	O^1^Asp63	O^2^Asp63	OTyr95	OTyr188	O^1^CO_3_	O^2^CO_3_
Al	2.06	1.86	3.98	1.83	1.91	2.01	1.98
Cr	2.04	1.79	3.90	1.78	1.79	1.90	1.95
Fe	1.99	1.82	3.89	1.85	1.85	1.90	1.84
Ga	2.08	1.95	4.01	1.91	2.07	2.00	2.13
In	2.26	2.13	4.13	2.11	2.23	2.19	2.25

#### 2. Chelators interaction with aluminum

**Fig. 3:**
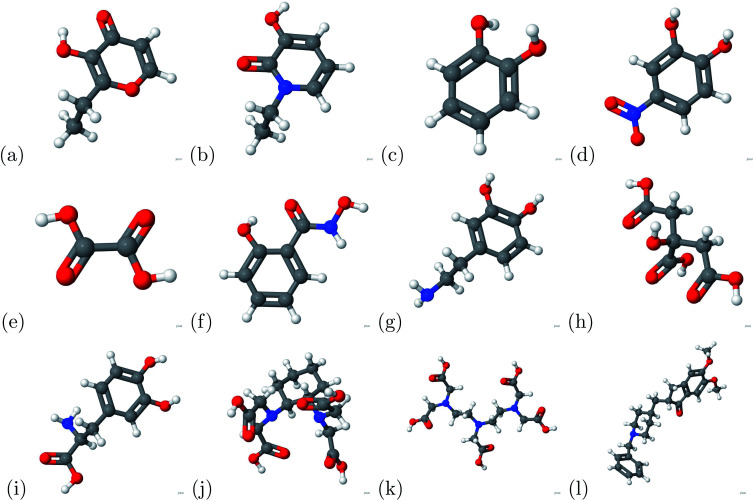
The chelators above were used in the current study. (a) 2-Ethyl-3-hydroxypyr-4-one, (b) 1-ethyl-3-hydroxypyridin-2-one, (c) catechol, (d) 4-nitro-catechol, (e) oxalic acid, (f) salicylhydroxamic acid, (g) dopamine, (h) citric acid, (i) l-Dopa, (j) CDTA, (k) DTPA and (l) donepezil. For the atomic structure, the carbon, hydrogen, oxygen, nitrogen, and aluminum atoms are represented by gray, white, red, blue, and grayish red respectively.

Due to aluminum's high affinity for human serum transferrin and its pathophysiology, we proceeded to investigate potential chelators. The molecules of the chelators used in this study are presented in Fig. 3: (a) 2-ethyl-3-hydroxypyr-4-one, (b) 1-ethyl-3-hydroxypyridin-2-one, (c) catechol, (d) 4-nitro-catechol, (e) oxalic acid, (f) salicylhydroxamic acid, (g) dopamine, (h) citric acid, (i) l-Dopa, (j) CDTA, (k) DTPA and (l) donepezil. Total energy calculations were conducted to determine the binding affinity based on eqn (1). A large negative magnitude binding energy indicates a strong bond. Furthermore, if a charge transfer is present, there is indication of a strong bond based on eqn (2). The successful chelators were determined by comparing the binding energy between the aluminum ion and chelator to the aluminum ion and transferrin complex.

### C. Binding energy

**Fig. 4:**
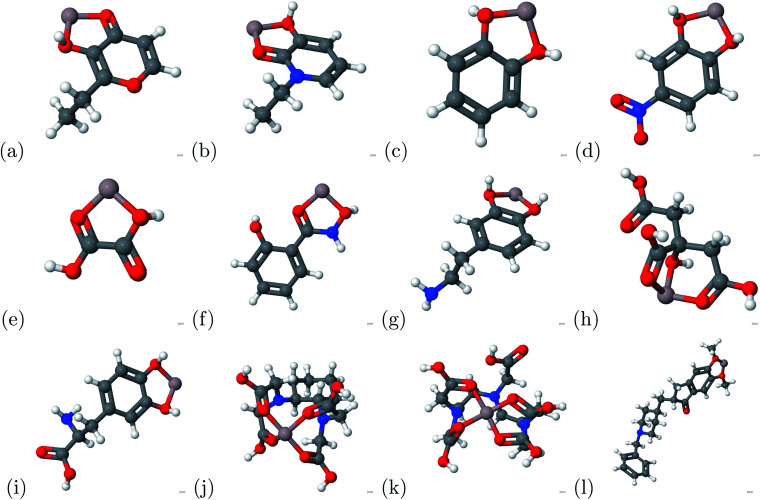
The atomic structures of the aluminum–chelator complexes are shown above. (a) Aluminum–2-ethyl-3-hydroxypyr-4-one complex, (b) aluminum–1-ethyl-3-hydroxypyridin-2-one complex, (c) aluminum–catechol complex, (d) aluminum–4-nitro-catechol complex, (e) aluminum–oxalic acid complex, (f) aluminum–salicylhydroxamic acid complex, (g) aluminum–dopamine complex, (h) aluminum–citric acid complex, (i) aluminum–l-Dopa complex, (j) aluminum–CDTA complex, (k) aluminum–DTPA complex and (l) aluminum–donepezil complex. For the atomic structure, the carbon, hydrogen, oxygen, nitrogen, and aluminum atoms are represented by gray, white, red, blue, and grayish red respectively.

The binding energy was calculated based on eqn (1). The binding energy was used to determine potential chelators of aluminum from the human serum transferrin binding site. If the binding energy was similar or larger than the aluminum–transferrin binding energy then it could be a potential chelator. The binding energies between aluminum and potential chelators are presented in Table 4. The binding interactions are presented in Fig. 4. From the calculations conducted during this study, 6 of the 12 chelators studied have a strong and stable bond with aluminum: 1-ethyl-3-hydroxypyridin-2-one, CDTA, citric acid, DTPA, oxalic acid, and salicylhydroxamic acid. The absolute difference between the aluminum–human serum transferrin complex and the 6 chelators above are 57.1269 kcal mol^−1^, 0.5386 kcal mol^−1^, 56.7726 kcal mol^−1^, 32.3506 kcal mol^−1^, 45.1727 kcal mol^−1^, and 60.3557 kcal mol^−1^ respectively. Because this is a very minimal energy difference and the metal uptake and release mechanisms were not studied further research is still needed to determine the effectiveness of these chelators.

**Table 4:** Binding energy (kcal mol^−1^) between aluminum and potential chelators

Chelator	*E*_binding_ (kcal mol^−1^)
2-Ethyl-3-hydroxypyr-4-one	1.0016
1-Ethyl-3-hydroxypyridin-2-one	−3.7871
Catechol	24.3887
CDTA	−61.4526
Citric acid	−4.1414
Donepezil	22.3943
Dopamine	22.7180
DTPA	−28.5634
l-Dopa	16.7724
4-Nitro-catechol	6.7562
Oxalic acid	−15.7412
Salicylhydroxamic acid	−0.5582

The binding energies between the metals and human serum transferrin are presented in Table 5. These values were then used to determine the indicator potential. Metals with similar binding energies with the iron–human serum transferrin complex can be potential indicators of AD.^26^ Gallium and indium have relatively similar binding energies to that of iron. Because of this, the use of radioactive gallium and indium have the potential to indicate early stages of AD. Further research is still necessary to determine the effectiveness of this treatment method.

**Table 5:** Binding energy (kcal mol^−1^) between metals and human serum transferrin's first coordination sphere

Metal	Al	Cr	Fe	Ga	In
*E*_binding_ (kcal mol^−1^)	−60.9140	−34.6876	−75.2027	−70.3894	−72.6776

### D. Charge transfer

**Fig. 5:**
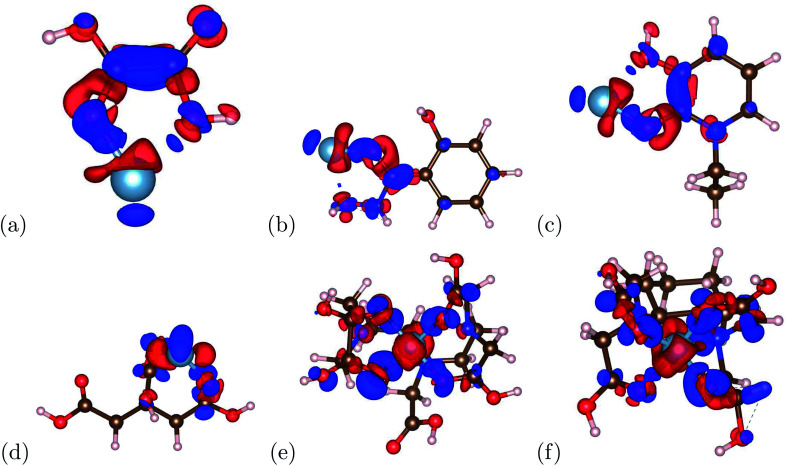
The charge transfers of the aluminum–chelator complexes are shown above. (a) Aluminum–oxalic acid complex, (b) aluminum–salicylhydroxamic acid complex, (c) aluminum–1-ethyl-3-hydroxypyridin-2-one complex, (d) aluminum–citric acid complex, (e) aluminum–DTPA complex and (f) aluminum–CDTA complex. For the atomic structure, the carbon, hydrogen, oxygen, nitrogen, and aluminum atoms are represented by brown, pink, red, blue, and light blue respectively. For the charge transfer, the red and blue denote positive isosurface (gain of electrons) and negative isosurfaces (loss of electrons) respectively.

The charge transfer calculations are based on eqn (2). Due to aluminum's high affinity for human serum transferrin and its pathophysiology, the charge transfer calculations were performed for the 6 chelators that showed a negative binding energy: oxalic acid, salicylhydroxamic acid, 1-ethyl-3-hydroxypyridin-2-one, citric acid, DTPA, and CDTA. The visualizations of the charge transfer are presented in Fig. 5. These 6 chelators displayed signs of a strong and stable bond.

## IV. Conclusion

We performed density functional theory calculations to determine the binding energy between different metals and human-serum transferrin to propose adequate indicators, and we also calculated the binding energy between aluminum and different chelators to propose solutions for chelation therapy.

Binding energy calculations were conducted for the aluminum chelator complexes portrayed in Fig. 4. The indicator potential was determined by comparing the iron–human serum transferrin binding energy with different metal–human serum transferrin binding energies. We found that the gallium–human serum transferrin complex and indium–human serum transferrin complex have similar binding energies with the iron–human serum transferrin complex. The absolute difference between the gallium–human serum transferrin complex and indium–human serum transferrin complex are 4.8134 kcal mol^−1^ and 2.5252 kcal mol^−1^. Previous experimental studies showed that gallium could be a potential indicator of Alzheimer's disease.^26^ This proves that further research is necessary due to the small absolute difference.

The chelation potential was determined by comparing the aluminum–human serum transferrin binding energy and the aluminum–chelator binding energies. We found that CDTA is the only chelator that has a higher binding energy than the binding energy of the aluminum–human serum transferrin complex. The absolute difference between the binding energies is −0.5386 kcal mol^−1^. Furthermore, CDTA has been seen to be an effective chelator of aluminum in previous experimental studies.^33^ The charge transfer showed that the 6 chelators have relatively strong bonds. Due to this small absolute difference and charge transfer calculations, further research is necessary to determine the effectiveness of the chelators. This is true for 1-ethyl-3-hydroxypyridin-2-one, citric acid, DTPA, oxalic acid, and salicylhydroxamic acid as well. Future research for these chelators may help with advances in chelation therapy for AD.

Due to computational limits, this study could only calculate the binding energy between the metal and the first coordination sphere of human serum transferrin. Future calculations and experiments may be useful to determine the true effectiveness. Future computations should consider this computational approach, but for the whole protein instead of only the first coordination sphere to provide an accurate determination of the binding energy so that we can determine the most optimal chelator for chelation therapy in AD patients. This study has provided 2 different indicators and 6 different chelators that prove to be capable for the indication of AD and the chelation of aluminum.

## Conflicts of interest

There are no conflicts of interest to declare.

## Supplementary Material
